# Engaging Family Physicians in the Provision of Palliative and End-of-Life Care: Can We Do Better?

**DOI:** 10.1089/pmr.2021.0021

**Published:** 2021-07-16

**Authors:** Tara McCallan, Helena Daudt

**Affiliations:** ^1^ Moss Rock Medical, Island Health Palliative and End of Life Program and Victoria Hospice, Victoria, British Columbia, Canada.; ^2^ UBC Faculty of Medicine, Vancouver, British Columbia, Canada.; ^3^ Department of Education and Research, Victoria Hospice, Victoria, British Columbia, Canada.; ^4^ School of Nursing, University of Victoria, Victoria, British Columbia, Canada.

**Keywords:** end-of-life care, family physicians, palliative care, primary care providers

## Abstract

***Background:*** Evidence shows the benefits of having a family physician (FP) at the heart of a care team that delivers palliative and end-of-life care (PEoLC). However, FPs have limitations on their ability to provide PEoLC.

***Objectives:*** We conducted a quality improvement study to (1) explore the barriers FPs encounter in providing PEoLC in our metropolitan context and (2) identify potential strategies to overcome these challenges.

***Methods:*** We interviewed a cohort of FPs from 10 different clinical practices within a metropolitan area (British Columbia [BC], Canada); this cohort is not regularly engaged with our Specialist Palliative Care Team. Verbatim transcripts were examined using inductive thematic analysis.

***Results:*** All FPs identified home visits as a critical aspect of being able to provide PEoLC. Despite this consensus, work-life balance, time, and compensation are major barriers to providing home visits and PEoLC. Local healthcare system awareness (available resources, why and how to access them) was identified as a barrier that can potentially be addressed through education sessions. Although 5 out of 10 FPs had not had formal palliative care education or training, clinical education was not considered a barrier to provide PEoLC.

***Conclusion:*** Providing FPs with tools and resources through education, including why and how to access them, and adjusting the BC compensation model to address home visit's travel time and time modifiers may better support FPs to provide PEoLC.

## Introduction

Research suggests that most people prefer to die at home rather than in a hospital setting.^1,2^ In Canada, 75% of people would choose to die at home.^3^ Ongoing recognition of this individual preference has resulted in federal policies that increasingly emphasize community-based care at end of life.^4,5^ In 2015, the Canadian Medical Association developed a policy including the recommendation that “all patients should have a primary care provider (PCP) that can support them with their palliative care needs or else refer these patients earlier to a palliative care team to establish goals of care.”^6^

Evidence highlights the benefits of having a PCP at the heart of a care team that delivers palliative and end-of-life care (PEoLC). The central involvement of a PCP improves the coordination of care,^7^ and studies indicate that fewer acute care services are used when a PCP anchors the chain of continuity of care.^8,9^

Despite their importance in delivering, sharing care, or referring patients to palliative care services, PCPs' capacity to engage in palliative care has been challenging. Carey et al.,^10^ in a recent systematic review, reported that lack of skills or confidence in managing symptoms, psychosocial aspects of care, bureaucratic procedures, communication with palliative care services and professionals, and personal and family commitments of the PCPs are important barriers to being engaged in PEoLC.

Previous national surveys have reported that 45% of Canadian family physicians (FPs) recognize the care they provide as palliative care, and only 42% of respondents provided home visits.^11^ Less than 40% of family medicine residents planned to include house calls as part of their practice.^12^ In the United States, the scenario is similar: only one-third of the FPs recertifying the America Board of Family Medicine Maintenance of Certification in 2013 saw themselves as palliative care providers, although it varied according to demographics and clinic settings.^13^

Few Canadian studies identified barriers to the involvement of PCPs in PEoLC, including gaps in knowledge (about the healthcare system and PEoLC) and the time required for home visits.^14–18^ In the United States, Kogan et al.,^19^ in the inaugural issue of Palliative Medicine Reports, pointed out that engaging PCPs to refer patients to a home-based palliative care service in northern California is complicated. They refer to lack of time, lack of palliative care health literacy, and the desire to retain oversight of their patient's care as important barriers.

Our study focused on an urban area in British Columbia (BC). FPs from this region are able to connect with a Specialist Palliative Care Team (SPCT) when providing PEoLC. Despite available access to this highly specialized team, the experience has been that many FPs from this particular area do not engage in PEoLC directly, nor do they collaborate with the SPCT.

## Methods

We interviewed FPs from 10 different clinical practices, who are not regularly engaged with the SPCT. The goals of our study were to (1) explore the barriers FPs encounter in providing PEoLC in our BC metropolitan context and (2) identify potential strategies to overcome these challenges. Our study qualifies as a quality improvement initiative and, therefore, did not require research ethics board review. It was registered with the Health Authority Quality Improvement Registry. Participants provided informed consent before being interviewed.

Following the framework proposed by Braun and Clarke,^20^ we examined verbatim transcripts of the interviews using thematic analysis. We used an inductive approach to this analysis. We also chose to provide a thematic description of our entire data set rather than a detailed account of one particular aspect of the data. Each author conducted a semantic analysis of the data and generated independent codes. Based on the number of times they were raised, codes were ranked by relevance to reach our goals. We then gathered to share our findings, discuss the identified codes, and reached consensus on themes. In conducting the semantic analysis, we recognized the need to perform a second level of analysis that would explore the underlying meanings of the collected data. In a second phase, we re-read the transcripts and identified one additional theme through latent analysis.

Data saturation^21^ (i.e., when no new code or new themes were identified) was reached after eight interviews. We conducted two additional interviews and confirmed data saturation as no new code or theme was identified.

## Results

All FPs interviewed in our study reported working full time and not sharing patients with other FPs. However, all but one (9 out of 10) reported participating in a practice group that covers for each other occasionally. Interviewed FPs practice in a wide geographical area within the Great Victoria region (Victoria, Oak Bay, Esquimalt, Saanich and Langford). Demographics data for participants are summarized in Table 1.

**Table 1.** Demographic Information for Study Participants

Participants (gender)	Completed family medicine training (year)	Family medicine training location (country)	Palliative care education	Current practice (days/week)
Participant 1 (man)	1985	Canada and United States	No formal training. Short lectures at Medical School +1-day Continuing Medical Education course	4.5
Participant 2 (man)	2015	Not mentioned	1-Month Palliative Care rotation (Residency)	4.5
Participant 3 (man)	1990	Canada	No formal training	4.5
Participant 4 (man)	2016	Canada	Geriatrics/Palliative Care rotation (Residency)	5
Participant 5 (man)	2007	Canada	Residency	3.5
Participant 6 (man)	2008	England	Family Practice Training (Hospice rotation)	5
Participant 7 (man)	1986	Canada and New Zealand	No formal training	7 half days
Participant 8 (woman)	2006	Canada	Palliative Care rotation (Residency)	4
Participant 9 (man)	1978	New Zealand	No formal training	4
Participant 10 (woman)	1983	Canada	No formal training	4

We identified five major barriers FPs encounter in providing PEoLC in our BC metropolitan context: time, work-life balance, compensation, communication with other healthcare providers, and role confusion.

All FPs identified home visits as a critical aspect of being able to provide PEoLC. Despite a consensus around the importance of providing this type of care to patients at their homes, some physicians raised challenges, which, in some cases, result in a complete inability to provide home visits. As one physician put it:

*I would like to contribute without being frustrated by the amount of time it takes. (…) If I can get to them easily, then yes I'll do home visits. (Participant 1)*

Work-life balance was another factor limiting FPs' availability for providing home visits. Some FPs commute by bicycle, which restricts their ability to visit patients who live far away from their office or home. Most FPs reported that they provided home visits after hours or on weekends.

Although one FP reported that *it doesn't even enter the equation for me* (Participant 10), many FPs in our study identified limited compensation as a major barrier. Some participants from our study argued that compensation for providing PEoLC does not align with compensation in other areas of care:

*I am very, very focused on my billing. Maximizing my billing, making sure billing's efficient and I'm strong believer that we are underpaid* (Participant 4).

*As office expenses go up, I find office absences to be increasingly expensive, so there are financial barriers to taking time to transport myself, way across town often to see a patient, for very little remuneration* (Participant 9).

Participants identified communication as an essential element in their ability to provide PEoLC. Communication with homecare nurses as well as with local palliative care resources was seen as an important part of routine practices, concurrent challenges, and potential opportunities to improve care. One physician cited communication gaps that limit knowledge sharing about what care is being provided to a patient in the community by other professionals:

*I wouldn't trust that I necessarily know […] which nurse is going, when they're going to go and that I'm going to have access to someone right away when I need them.* [Participant 8]

FPs generally valued feeling part of a team in the provision of PEoLC, yet a few reported some confusion about roles and responsibilities. We found this to be a recurrent barrier to the provision of PEoLC care: *I find that I'm not 100% sure what I'm responsible for* (Participant 3).

Although confusion about expectations proved a challenge to FPs, some physicians mentioned knowledge about available resources as an important opportunity to improve their ability to provide PEoLC: *if I were better informed about what the process is, […] it would be easier for me, and my patient* (Participant 1). Interestingly, participants did not name limited education as a barrier to provide PEoLC.

Since completing the interviews for this study, we piloted a “Dine & Learn” initiative. Local PCPs were invited to attend an evening event where members of the tertiary palliative care team shared processes and available resources (i.e., educational materials, clinical care processes, and human resources such as the crisis palliative care team). Consisting of FPs and nurse practitioners, 21 PCPs attended the event. Feedback received suggested that the event was successful in initiating connections between FPs, nurse practitioners, and the tertiary palliative care team, introducing useful resources/tools and, perhaps more importantly, why and how to access these local resources.

One additional theme emerged from the interview analysis, which was categorized as “Willingness…but.” The majority of FPs identified PEoLC as an intrinsic part of family medicine. FPs value the opportunity to contribute to a team providing care until death and are willing to support patients and families in this way. And yet, having learned from colleagues and by doing it, 5 out of 10 participants have had no formal palliative care education or training. FPs recognize many barriers that prevent them from providing PEoLC and practice family medicine in a way that meets their hopes and expectations.

## Discussion

Our study collected data from a small group of physicians practicing within a wide geographical area of our BC metropolitan region. Despite the small group size, we heard a consistent message from FPs, and so we are confident that it adequately represents the reality of our community. By collecting data through interviews we provided a venue for FPs to expand on their views, differing from many previously published articles that conducted quantitative surveys of a large number of professionals. Most of the barriers mentioned by the FPs we interviewed, however, matched very closely what has been described before: lack of system awareness, communication gaps among professionals, and challenges to provide home visits.

Our qualitative data provide interesting nuances to the challenges FPs experience to provide home visits. Maintaining their work-life balance (e.g., by commuting on bicycle or prioritizing time with their families) was an important factor limiting FPs' ability to provide home visits. Most FPs reported that they provided home visits after hours or on weekends. Spice et al.^15^ mentioned, in their Ontarian study, that provision of home visits during and after office hours was a major barrier in supporting people at end of life. Osborn et al.^22^ points out that work-life balance, including acceptable hours of practice and lifestyle flexibility, was an important factor influencing Canadian medical students to choose family medicine or pediatrics. It is possible that the prospect of maintaining a good work-life balance had been key to the career choice of the FPs participating in our study, perhaps causing some internal conflict and, therefore, affecting the way they practice PEoLC. In addition, compensation was a barrier highlighted by FPs, also linked to home visits, but not mentioned in other Canadian studies. Home visits in BC are paid as fee-for-service, do not include travel time or time-modifiers (FPs are paid the same for a home visit between 8 am and 11 pm, any day of the week and independent of the duration of the visit).^23^ The compensation of home visits in Ontario, for example, includes travel time and varies with time and day (daytime vs. evenings; weekdays vs. weekends).^24^ Changing the compensation models to address these differences may better support FPs to provide PEoLC in BC.

Even though previous studies do not report on the specific type of palliative care training that FPs receive, if any, Tan et al.^16^ and Malik et al.^17^ maintain that FPs from Ontario and Alberta value educational opportunities as a means to sustain and/or improve their skills and knowledge in this area. Half the participants in our study mentioned undertaking a hospice or palliative care rotation during family medicine training. Simmons et al.^25^ found that residents in a four-week palliative medicine rotation program recognized it as a highly valuable component of their training and reported significant improvements in their level of comfort in all areas of measured end-of-life care. Conversely, half of the participants in our study reported that they had no formal palliative care education. Participants did not name limited education as a barrier, but they did identify learning about available resources and improving communication with the interdisciplinary team as an opportunity to improve their ability to provide PEoLC. The Dine & Learn educational initiative addressed these two topics. Although conducting a long-term post-intervention evaluation was beyond the scope of the initiative, feedback received at the event indicated that it was successful to achieve these goals. Kelley et al.^26^ highlight interprofessional collaboration as one of seven education topics addresses through education that suggests improvement in FPs' perceived attitudes, confidence, knowledge, and skills to provide PEoLC.

Tan et al.^27^ recently provided a conceptual framework portraying many practical aspects to improve the patient and caregiver palliative journey by fostering team relationships between all care providers and building on the trusting FP-patient longitudinal relationship. Upon completion of this exploratory study and as work was underway on this report, additional publications about the theme became available complementing our findings.^10,19,26,27^ In a recent article in *The Ottawa Citizen*, Booke and Stajduhar stated, “As Canadians, we now have a legal right to medical assistance in dying. It's time to demand that we should have an equal right to medical assistance in living.”^28^ We would argue that our Canadian healthcare system can better support medical assistance in living by ensuring that FPs have access to education that includes available PEoLC tools and resources, how and why to access those resources and focuses on interprofessional communication, and by providing fair compensation that addresses the challenges of conducting home visits.

## Abbreviations Used

BC: British Columbia
FP: family physician
PCP: primary care provider
PEoLC: palliative and end-of-life care
SPCT: Specialist Palliative Care Team
